# 178. Elevating Infectious Diseases Management: Impact of an Inpatient Pharmacist-Led Tele-Antimicrobial Stewardship Program Integrated within a Health System

**DOI:** 10.1093/ofid/ofae631.058

**Published:** 2025-01-29

**Authors:** Bejoy Paul Maniara, Jomi Oommen, Jeffery Thomas, Catherine Millares-Sipin

**Affiliations:** Northwell Health, Syosset, NY; Northwell Health, Syosset, NY; Northwell Health, Syosset, NY; Northwell Health, Syosset, NY

## Abstract

**Background:**

Antimicrobial resistance (AR) is a growing global threat projected to cause 10 million deaths annually by 2050. Joint Commission requires hospitals to have antimicrobial stewardship programs (ASPs). Centers for Disease Control and Prevention recommends pharmacists co-lead ASPs. Infectious diseases (ID) pharmacists are critical to combat AR and elevate patient care by optimizing antimicrobial use. Tele-ASPs could enhance patient care by extending patient care continuity. This study assesses the impact of an inpatient pharmacist-led Tele-ASP.

**Figure:**
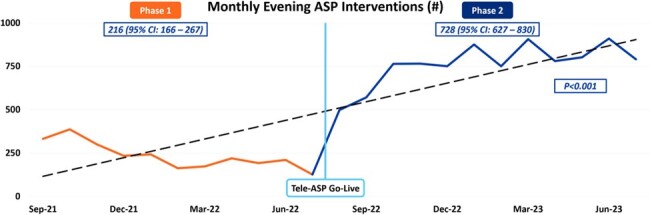

This chart depicts monthly ASP-related pharmacist interventions, comparing the amount performed before and after implementation of the Tele-ASP.

**Methods:**

This study evaluated antimicrobial management across 14 Northwell Health hospitals in two phases: Pre-Tele-ASP (August 15, 2021 – August 14, 2022) and Post-Tele-ASP (August 15, 2022 – August 14, 2023). The primary endpoint was the total number of ASP interventions during Tele-ASP hours (i.e., 4 PM – 12 AM). Secondary endpoints included ASP interventions and acceptance rates, cost savings, and length of stay (LOS).

**Figure:**
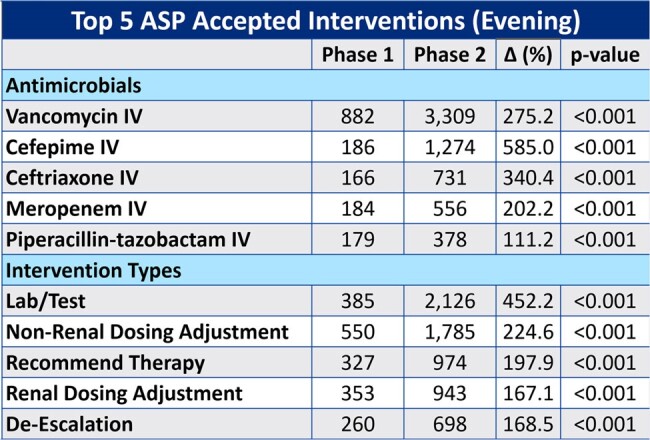

This chart outlines the top 5 antimicrobials that pharmacists performed ASP-related interventions on, as well as the top 5 ASP-related interventions that were performed.

**Results:**

A total of 19,802 patients with ASP interventions were included; 5,919 patients (29.9%) had interventions during Tele-ASP hours. There was a 237% increase in ASP interventions during operating hours in the post-Tele-ASP phase (9,467 vs 2,810; p < 0.001). Overall, there was a 62% increase in accepted ASP interventions (25,192 vs 15,552; p < 0.001), with a 26% increase during the day shift (15,423 vs 12,257; p < 0.001), a 256% increase during the evening shift (9,248 vs 2,601; p < 0.001), and a 25% decrease during the overnight shift (524 vs 694; p < 0.01). The overall ASP intervention acceptance rate was 96.8%. Total cost savings and avoidance across all shifts increased by 74.1% ($10,355,707 vs $5,947,578; p < 0.001). The overall LOS decreased by a mean of 1.1 days (p < 0.001).

**Figure:**
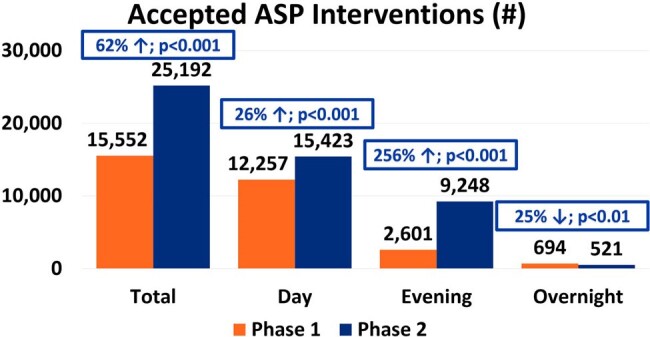

This chart stratifies the total number of accepted ASP interventions across each shift.

**Conclusion:**

This unique pharmacist-led Tele-ASP provided services to hospitals across Northwell Health during resource-limited hours and locations. Implementation increased accepted ASP-related interventions without reducing overall interventions, effectively reducing LOS and increasing cost savings and avoidance. This model serves as a blueprint for hospitals to strategically expand ASP services in a clinically and financially efficient manner and optimize antimicrobial use and patient care.

**Figure:**
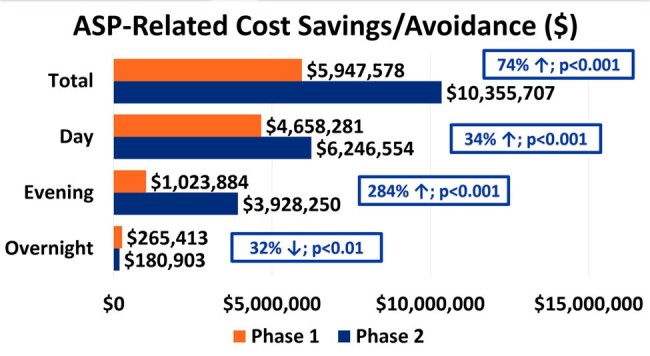

This chart stratifies the total amount of cost savings and cost avoidance, as a result of ASP-related pharmacy interventions, across each shift.

**Disclosures:**

**All Authors**: No reported disclosures

